# Association between dietary multi-metal intake and the risk of diabetic retinopathy: a population-based study

**DOI:** 10.3389/fnut.2025.1595788

**Published:** 2025-06-19

**Authors:** Chaohua Zhang, Haiyang Peng, Qin Lang, Haoyu Fang, Keqin Zhang, Andong Zhao

**Affiliations:** ^1^Department of Urinary Nephropathy Center, Second Hospital Affiliated to Chongqing Medical University, Chongqing, China; ^2^Department of Hepatobiliary Surgery, Second Hospital Affiliated to Chongqing Medical University, Chongqing, China; ^3^Department of Clinical Nutrition, Chongqing Red Cross Hospital (People's Hospital of Jiangbei District), Chongqing, China; ^4^Department of Plastic and Maxillofacial Surgery, Second Hospital Affiliated to Chongqing Medical University, Chongqing, China

**Keywords:** diabetic retinopathy, dietary metals intake, weighted quantile sum, Bayesian kernel machine regression, NHANES

## Abstract

**Objective:**

To investigate the association between dietary metals intake and the risk of diabetic retinopathy (DR) in adults with diabetes.

**Methods:**

Data from 2,822 U. S. adults with diabetes in National Health and Nutrition Examination Survey (NHANES) 2007–2016 were analyzed. Associations between the intake of six dietary metals and DR risk were assessed using multivariable logistic regression, Weighted Quantile Sum (WQS) regression, and Bayesian Kernel Machine Regression (BKMR). Restricted Cubic Spline (RCS) regression examined the dose–response relationship between intake of dietary metal and DR risk. Mediation analysis explored the underlying mechanisms.

**Results:**

Log10-transformed dietary Zinc (Zn) (OR = 0.53, 95% CI 0.35–0.80, *p* = 0.003) were negatively associated with the DR risk. WQS regression indicated that the combined effects of dietary metals intake were negatively associated with the risk of DR (OR = 0.79, 95% CI 0.61–0.97, *p* = 0.024), with Zn contributing the most to the reduced risk (36.4%). BKMR model suggested the negative association between the combined intake of 6 metals and DR risk, with Zn receiving the highest posterior inclusion probability (PIP) (0.8574).

**Conclusion:**

In American adults with diabetes, elevated dietary metals intake, especially zinc, may be associated with a lower risk of DR.

## 1 Introduction

Diabetic microvascular complications significantly threaten the quality of life in individuals with diabetes, with retinopathy being the most prevalent manifestation (1). Numerous studies have consistently identified smoking, alcohol consumption, physical inactivity, and hypertension as major risk factors for diabetic retinopathy (DR) (2, 3). DR is pathologically characterized by progressive retinal damage, primarily mediated through inflammation and oxidative stress, leading to irreversible structural and functional alterations (4, 5). Early-stage DR is generally asymptomatic, making early detection difficult. As DR progresses to advanced stages, it can cause irreversible vision loss and impose substantial therapeutic and socioeconomic burdens (6). Therefore, it is necessary to identify modifiable factors for preventing DR among people with diabetes.

The intake of dietary metals including zinc (Zn), copper (Cu), magnesium (Mg), iron (Fe), calcium (Ca), and selenium (Se), is biologically essential, as these elements collectively participate in enzymatic activation, hemoglobin synthesis, antioxidant defense, bone mineralization, and immune regulation (7, 8). Beyond their essential physiological functions, growing evidence suggests an association between various dietary metals and diabetes risk. In a median 9-year follow-up, He P et al. demonstrated a U-shaped association between dietary Zn intake and incident diabetes, with an inflection point at approximately 9.1 mg/day (9). A prospective cohort study from China found that adequate Fe intake (>23 mg/day) may help prevent diabetes, while excessive Fe intake (>46 mg/day) may increase the risk of diabetes in males (10). Additionally, a cross-sectional study of US adults demonstrated an inverse relationship between dietary Mg intake and diabetes occurrence (OR: 0.56, P trend < 0.001) (11). While these findings clarify the role of dietary metals in diabetes onset, their impact on complications, particularly retinopathy progression, remains underexplored, creating a critical knowledge gap in dietary metals-based complication prevention strategies.

To better understand this relationship between dietary metal intake and DR, we conducted a cross-sectional study using data from the National Health and Nutrition Examination Survey (NHANES) 2007–2016. We applied three regression models to assess both individual and combined associations of six dietary metals with DR risk and to determine the relative importance of each metal. This study may provide population-based evidence on the potential protective role of dietary metals intake against DR, with a particular emphasis on zinc.

## 2 Materials and methods

### 2.1 Study participants

The NHANES, conducted by the National Center for Health Statistics (NCHS), employs stratified multistage sampling to collect nationally representative data on health and nutritional status among US civilians (12). The original protocols received NCHS Ethics Review Board approval with documented informed consent from all participants. As this study involved secondary analysis of the NHANES public data (available at https://wwwn.cdc.gov/nchs/nhanes/default.aspx), no additional ethical review was required.

**Figure 1:**
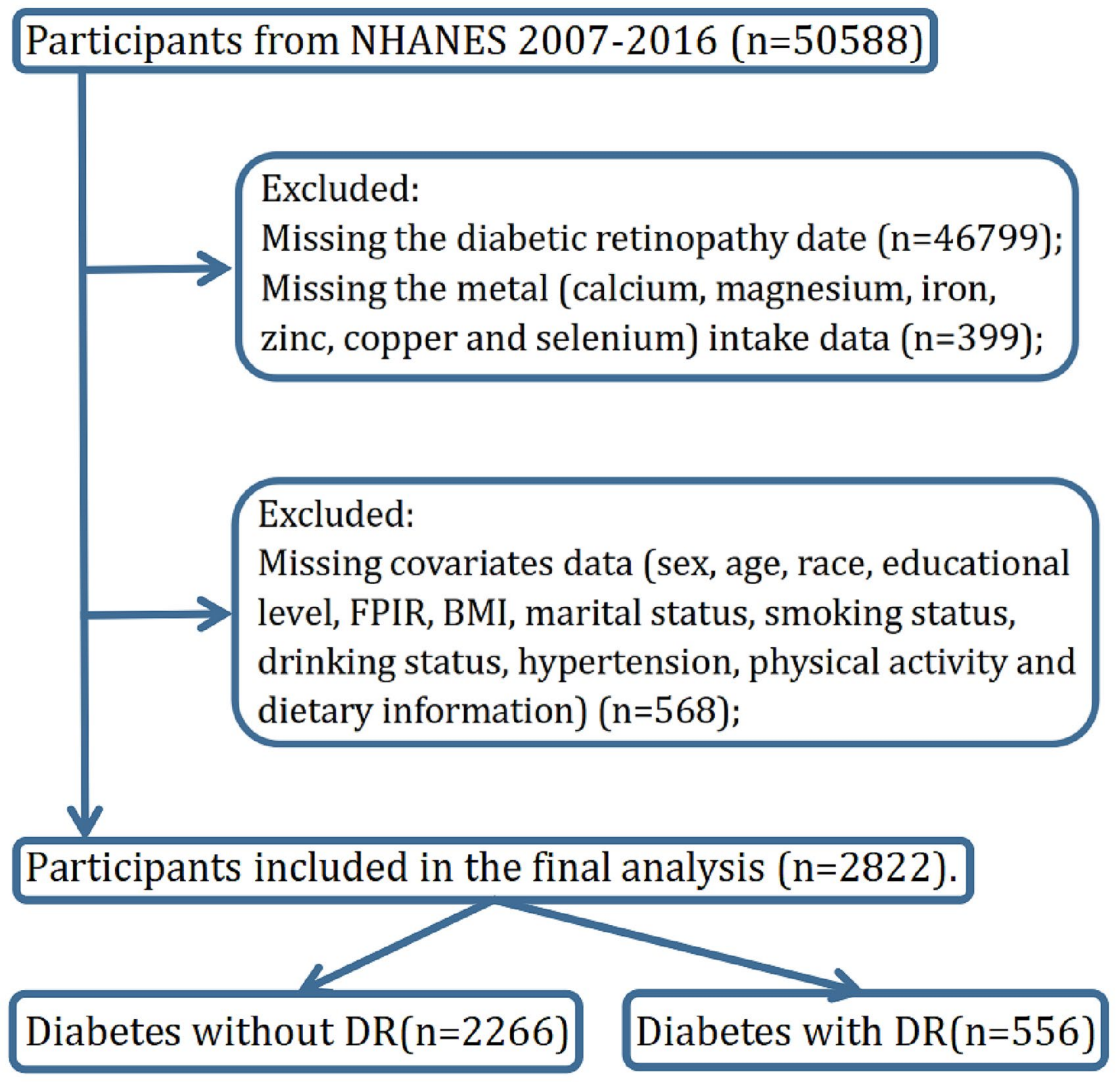
Identified participants though a flow chart in the study.

The individuals in this study were included from NHANES 2007–2016 (*n* = 50,588). Participants with missing DR assessment data were excluded (*n* = 46,799). The Diabetes Questionnaire (DIQ) item DIQ080, administered only to individuals with diabetes to assess if diabetes has affected the eyes or caused retinopathy, categorized recorded answers as valid (‘Yes’/'No’) or missing (‘Refused’/'Do not know’/'Missing’). Furthermore, participants lacking dietary metal intake data were excluded from the analysis (*n* = 399). Additionally, participants with no or invalidated data on covariates required for subsequent analyses were also excluded (*n* = 568), including sex, age, race, educational level, family poverty-income ratio (FPIR), body mass index (BMI), marital status, smoking status, drinking status, hypertension, physical activity, and dietary information. Finally, a total of 2,822 individuals with diabetes were included in this study analysis with 556 individuals with diabetes diagnosed with DR (Figure 1).

### 2.2 Confirmation of DR

In the questionnaire data of NHANES, the diabetes section (prefix DIQ) encompasses data obtained through personal interviews covering diabetes, prediabetes, insulin/oral hypoglycemic drug use, and diabetic retinopathy. It also incorporates self-reported accounts of diabetes risk factor awareness along with associated medical treatments and personal care routines. In this study, participants were determined to have DR if their physicians informed them that diabetes had affected their eyes or that they had retinopathy (13).

### 2.3 Assessment of dietary metals

In each NHANES cycle, participants submitted comprehensive dietary records across two non-consecutive 24-h periods, enabling estimation of energy expenditure, nutrient absorption, and food constituent intake. Initial dietary interviews were conducted face-to-face during examinations, with subsequent telephone-based recalls performed 3–10 days thereafter. For these analyses, the average intake of food-derived metals (Zn, Cu, Mg, Fe, Ca, and Se) was calculated over the two recall periods; if only the first day’s data was available, that figure was used instead of an average. Participants were also asked about their supplement use during the same two 24-h periods, and the intake of metals from supplements was averaged over the 2 days if possible. The dietary metals intake was determined by summing both supplement and food-derived sources.

### 2.4 Covariates and mediators

Various sociodemographic information incorporating sex, age, race, educational level, FPIR, and marital status were comprehensively documented. The FPIR, derived from the ratio of family income to poverty thresholds adjusted for family size, year, and state of residence, utilized the Department of Health and Human Services’ poverty criteria and was categorized into three groups: low (≤1), middle (>1 to ≤4), and high (>4) (14). The smoking and drinking status were categorized as never, former, and current based on previous study (15). BMI (kg/m2) was calculated as weight divided by height squared. Hypertension was identified as having a systolic blood pressure of >130 mm Hg or diastolic blood pressure of >80 mm Hg from an average of 3 measurements or a history of high blood pressure or a history of oral antihypertensive medications (16). Physical activity, gained by the Global Physical Activity Questionnaire (17), was categorized into two groups based on whether participants met the 2018 physical activity guidelines (18). Detailed records of protein, total fat, carbohydrate, dietary fiber, and energy intake derived from foods and supplement consumption were obtained using two 24-h dietary recall administrations. Bilirubin (BIL), albumin (ALB), and *γ*-glutamyl transferase (GGT) were designated as oxidative stress indicators, while C-reactive protein (CRP), white blood cell (WBC), lymphocyte count, absolute neutrophil count, and alkaline phosphatase (ALP) served as inflammatory markers. These parameters have been extensively applied in NHANES research for quantifying oxidative stress and inflammatory responses (19–22).

### 2.5 Statistical analysis

Baseline characteristic data were grouped by diabetic retinopathy (DR) status. Continuous variables were presented means with standard deviation (SD) and categorical variables as frequencies with percentages. Group comparisons were conducted using *t*-tests for continuous variables and chi-squared tests for categorical variables.

The intake of dietary metals was log10-transformed due to skewed distributions then divided into quartiles. Multivariable logistic regression was utilized to evaluate associations between dietary metal intakes and DR prevalence. Three hierarchical models were constructed: Model 1 was adjusted for sex, age, race, educational level, and marital status; Model 2 further adjusted for FPIR, BMI, smoking status, drinking status, and hypertension; Model 3 further adjusted for physical activity, protein, total fat, carbohydrate, fiber, and energy intake. The first quartile (Q1) was set as the control group.

To assess the combined effects of the six dietary metals on DR prevalence, a WQS regression was applied by calculating a weighted linear index and assigning corresponding weights. A 1000-time bootstrapping procedure was used to construct WQS indices in both positive and negative directions. When the WQS index was significantly nonzero, corresponding weights were calculated to identify the relative contribution of each dietary metal within the index to the incidence of DR. The dataset was randomly split into training set (40%) and validation set (60%) according to previous research (22).

Additionally, the Bayesian variable selection framework was also utilized to explore the overall effects of six dietary metals mixtures on the odds of DR. To be specific, the BKMR model assessed the overall impact of mixtures levels at specific quartiles compared to the medians. All metal intakes were log10-transformed and standardized before analysis. The posterior inclusion probability (PIP) was calculated to quantify the relative importance of each metal (PIP > 0.5 indicating strong contribution). Moreover, univariate and bivariate exposure-response functions were applied to assess both the individual effects and interactions of dietary metals, while simultaneously considering the other dietary metals at the 25th, 50th, and 75th percentiles. This model conducted 10,000 iterations via the Markov Chain Monte Carlo algorithm after accounting for all covariates.

Restricted Cubic Spline (RCS) regression was conducted to examine potential dose–response relationships between log10-transformed dietary Zn intake and DR incidence (23). Subgroup analyses were performed stratified by age, sex, race, educational level, FPIR, marital status, smoking status, drinking status, BMI, hypertension, and physical activity.

Linear regression models were used to assess the associations between log10-transformed dietary Zn intake and the biomarkers of inflammation and oxidative stress. Mediation analysis was performed to estimate direct and indirect effects of dietary Zn on DR risk via inflammatory and oxidative stress pathways, with 1,000 bootstraps after adjusting all covariates.

In this study, data were not weighted as adjustments for demographic factors were already made (24). The statistical significance level was set at a *p* value of < 0.05. *R 4.4.2* software was utilized for all statistical analyses.

## 3 Results

### 3.1 Characteristics of participants

After excluding ineligible participants, a total of 2,822 individuals remained for further analysis. All included participants had diabetes, and 556 of them were diagnosed with DR. Among the qualified participants, As shown in Table 1, there were significant differences in sex, race, educational level, family poverty income ratio (FPIR), hypertension, and physical activity among the qualified participants (all *p* < 0.05).

**Table 1:** Baseline characteristics by diagnosis of DR in the NHANES 2007–2016 diabetic population.

Characteristic	Overall	Diabetes with DR		*p* value
		No (*N* = 2,266)	Yes (*N* = 556)	
Sex				0.009
Male	1,451 (51.42%)	1,137 (50.18%)	314 (56.47%)	
Female	1,371 (48.58%)	1,129 (49.82%)	242 (43.53%)	
Age (years)	61.18 ± 12.97	61.14 ± 13.08	61.34 ± 12.50	0.738
Race				0.037
Mexican American	498 (17.65%)	407 (17.96%)	91 (16.37%)	
Non-Hispanic Black	758 (26.86%)	599 (26.43%)	159 (28.60%)	
Non-Hispanic White	1,039 (36.82%)	858 (37.86%)	181 (32.55%)	
Other Hispanic	305 (10.81%)	232 (10.24%)	73 (13.13%)	
Other Race	222 (7.87%)	170 (7.50%)	52 (9.35%)	
Educational level				0.033
Less than college	1,620 (57.41%)	1,278 (56.40%)	342 (61.51%)	
Some college or above	1,202 (42.59%)	988 (43.60%)	214 (38.49%)	
FPIR	2.24 ± 1.51	2.28 ± 1.51	2.08 ± 1.51	0.005
Marital status				0.850
Live alone	1,165 (41.28%)	933 (41.17%)	232 (41.73%)	
Live together	1,657 (58.72%)	1,333 (58.83%)	324 (58.27%)	
Smoking status				0.408
Current	457 (16.19%)	374 (16.50%)	83 (14.93%)	
Former	973 (34.48%)	769 (33.94%)	204 (36.69%)	
Never	1,392 (49.33%)	1,123 (49.56%)	269 (48.38%)	
Drinking status				0.671
Current	1799 (63.75%)	1,453 (64.12%)	346 (62.23%)	
Former	507 (17.97%)	405 (17.87%)	102 (18.35%)	
Never	516 (18.28%)	408 (18.01%)	108 (19.42%)	
BMI	32.74 ± 7.60	32.71 ± 7.55	32.88 ± 7.83	0.648
Hypertension				0.001
No	419 (14.85%)	362 (15.98%)	57 (10.25%)	
Yes	2,403 (85.15%)	1904 (84.02%)	499 (89.75%)	
Physical activity reached				0.001
No	1,601 (56.73%)	1,250 (55.16%)	351 (63.13%)	
Yes	1,221 (43.27%)	1,016 (44.84%)	205 (36.87%)	
Energy from Diet (kcal/day)	1804.39 ± 753.66	1801.25 ± 730.02	1817.18 ± 843.79	0.682
Protein from Diet (g/day)	75.62 ± 33.97	75.33 ± 33.25	76.79 ± 36.77	0.395
Total fat from Diet (g/day)	71.96 ± 37.48	72.15 ± 37.09	71.16 ± 39.02	0.588
Carbohydrate from Diet (g/day)	212.01 ± 90.77	211.31 ± 89.09	214.88 ± 97.38	0.431
Dietary fiber from Diet (g/day)	16.03 ± 8.59	16.06 ± 8.49	15.92 ± 8.97	0.746

### 3.2 Dietary metals intake and DR risk in logistic regression model

To assess the relationship between six dietary metals intake and the odds of DR among individuals with diabetes, we employed univariate and multivariate logistic regression. As shown in Table 2, an inverse relationship between log10-transformed dietary Zn and Cu intake and the risk of DR was consistently observed across the crude model, model 1, model 2, and model 3. In model 3, the increase in log10-transformed dietary Zn (OR = 0.53, 95% CI 0.35–0.80, *p* = 0.003) and Cu (OR = 0.52, 95% CI 0.32–0.82, *p* = 0.005) intake was significantly correlated with a decreased prevalence of DR. Furthermore, when participants were categorized by the quartiles of log10-transformed dietary metal intake, individuals in the highest quartile group of dietary Zn intake (OR_Q4 vs. Q1_ = 0.62, 95% CI 0.45–0.86, *p* = 0.004, *p* for trend = 0.005) and dietary Cu intake (OR_Q4 vs. Q1_ = 0.63, 95% CI 0.45–0.88, *p* = 0.007, *p* for trend = 0.01) had a lower risk of DR compared to those in the lowest quartile group through logistic regression model 3. The rest of the four dietary metals intake had no significance to the occurrence of DR in individuals with diabetes.

**Table 2:** Multivariate logistic regression analysis of Log10-transformed metals intake for the prevalence of DR in diabetic population.

	Crude model		Model 1		Model 2		Model 3	
	OR (95% CI)	*p*-value	OR (95% CI)	*p*-value	OR (95% CI)	*p*-value	OR (95% CI)	*p*-value
Log10 Ca	0.80 (0.55, 1.16)	0.240	0.91 (0.62, 1.35)	0.636	0.91 (0.61, 1.34)	0.620	0.91 (0.58, 1.42)	0.667
Q1	Reference		Reference		Reference		Reference	
Q2	0.96 (0.74, 1.25)	0.772	0.99 (0.76, 1.29)	0.962	1.00 (0.77, 1.30)	0.994	0.99 (0.76, 1.30)	0.957
Q3	0.93 (0.71, 1.20)	0.568	0.97 (0.74, 1.26)	0.801	0.96 (0.73, 1.25)	0.757	0.97 (0.73, 1.29)	0.847
Q4	0.88 (0.68, 1.15)	0.356	0.96 (0.73, 1.26)	0.758	0.96 (0.73, 1.26)	0.772	0.97 (0.71, 1.32)	0.845
*p* for trend	0.335		0.722		0.714		0.822	
Log10 Cu	0.60 (0.42, 0.87)	**0.007**	0.60 (0.40, 0.87)	**0.008**	0.60 (0.41, 0.89)	**0.011**	0.52 (0.32, 0.82)	**0.005**
Q1	Reference		Reference		Reference		Reference	
Q2	0.85 (0.66, 1.10)	0.220	0.86 (0.66, 1.11)	0.250	0.87 (0.67, 1.13)	0.294	0.81 (0.61, 1.07)	0.139
Q3	0.87 (0.67, 1.12)	0.274	0.87 (0.66, 1.13)	0.282	0.88 (0.67, 1.14)	0.329	0.78 (0.56, 1.07)	0.123
Q4	0.70 (0.53, 0.91)	0.007	0.69 (0.53, 0.91)	0.010	0.70 (0.53, 0.93)	0.013	0.63 (0.45, 0.88)	0.007
*p* for trend	**0.012**		**0.015**		**0.019**		**0.010**	
Log10 Fe	0.94 (0.67, 1.31)	0.706	0.97 (0.69, 1.36)	0.861	0.99 (0.70, 1.39)	0.943	0.99 (0.67, 1.44)	0.958
Q1	Reference		Reference		Reference		Reference	
Q2	0.82 (0.63, 1.06)	0.129	0.81 (0.62, 1.06)	0.123	0.82 (0.63, 1.07)	0.152	0.82 (0.61, 1.09)	0.167
Q3	0.89 (0.69, 1.16)	0.393	0.89 (0.68, 1.16)	0.377	0.89 (0.68, 1.16)	0.396	0.85 (0.62, 1.16)	0.293
Q4	0.87 (0.67, 1.13)	0.297	0.88 (0.68, 1.15)	0.352	0.89 (0.68, 1.16)	0.382	0.85 (0.62, 1.16)	0.316
*p* for trend	0.441		0.502		0.522		0.388	
Log10 Mg	0.71 (0.45, 1.12)	0.140	0.69 (0.42, 1.11)	0.129	0.70 (0.43, 1.14)	0.153	0.60 (0.31, 1.16)	0.131
Q1	Reference		Reference		Reference		Reference	
Q2	1.15 (0.89, 1.49)	0.274	1.17 (0.90, 1.51)	0.239	1.18 (0.91, 1.53)	0.217	1.11 (0.84, 1.47)	0.465
Q3	0.86 (0.66, 1.13)	0.283	0.87 (0.66, 1.14)	0.322	0.89 (0.67, 1.17)	0.388	0.80 (0.57, 1.12)	0.188
Q4	0.88 (0.67, 1.14)	0.336	0.86 (0.65, 1.14)	0.296	0.87 (0.65, 1.15)	0.330	0.78 (0.53, 1.13)	0.191
*p* for trend	0.113		0.102		0.123		0.082	
Log10 Se	1.14 (0.75, 1.74)	0.531	1.08 (0.69, 1.67)	0.746	1.10 (0.70, 1.71)	0.684	1.10 (0.63, 1.94)	0.742
Q1	Reference		Reference		Reference		Reference	
Q2	0.89 (0.68, 1.16)	0.387	0.89 (0.68, 1.16)	0.389	0.90 (0.69, 1.18)	0.443	0.88 (0.66, 1.17)	0.380
Q3	0.98 (0.75, 1.27)	0.852	0.96 (0.74, 1.26)	0.777	0.97 (0.74, 1.27)	0.842	0.91 (0.65, 1.27)	0.589
Q4	1.04 (0.80, 1.34)	0.792	0.99 (0.75, 1.31)	0.958	0.99 (0.75, 1.31)	0.957	0.94 (0.66, 1.35)	0.754
*p* for trend	0.638		0.899		0.903		0.822	
Log10 Zn	0.61 (0.44, 0.85)	**0.003**	0.61 (0.43, 0.86)	**0.005**	0.62 (0.43, 0.87)	**0.006**	0.53 (0.35, 0.80)	**0.003**
Q1	Reference		Reference		Reference		Reference	
Q2	0.80 (0.62, 1.04)	0.093	0.80 (0.62, 1.04)	0.097	0.81 (0.63, 1.05)	0.119	0.74 (0.55, 0.98)	0.035
Q3	0.81 (0.63, 1.05)	0.107	0.80 (0.61, 1.04)	0.098	0.80 (0.61, 1.04)	0.098	0.69 (0.50, 0.95)	0.022
Q4	0.69 (0.53, 0.90)	0.007	0.70 (0.53, 0.92)	0.010	0.70 (0.53, 0.93)	0.013	0.62 (0.45, 0.86)	0.004
*p* for trend	**0.011**		**0.015**		**0.017**		**0.005**	

Bold indicates *p* < 0.05.

### 3.3 Dietary metals intake and DR risk in WQS model

**Figure 2:**
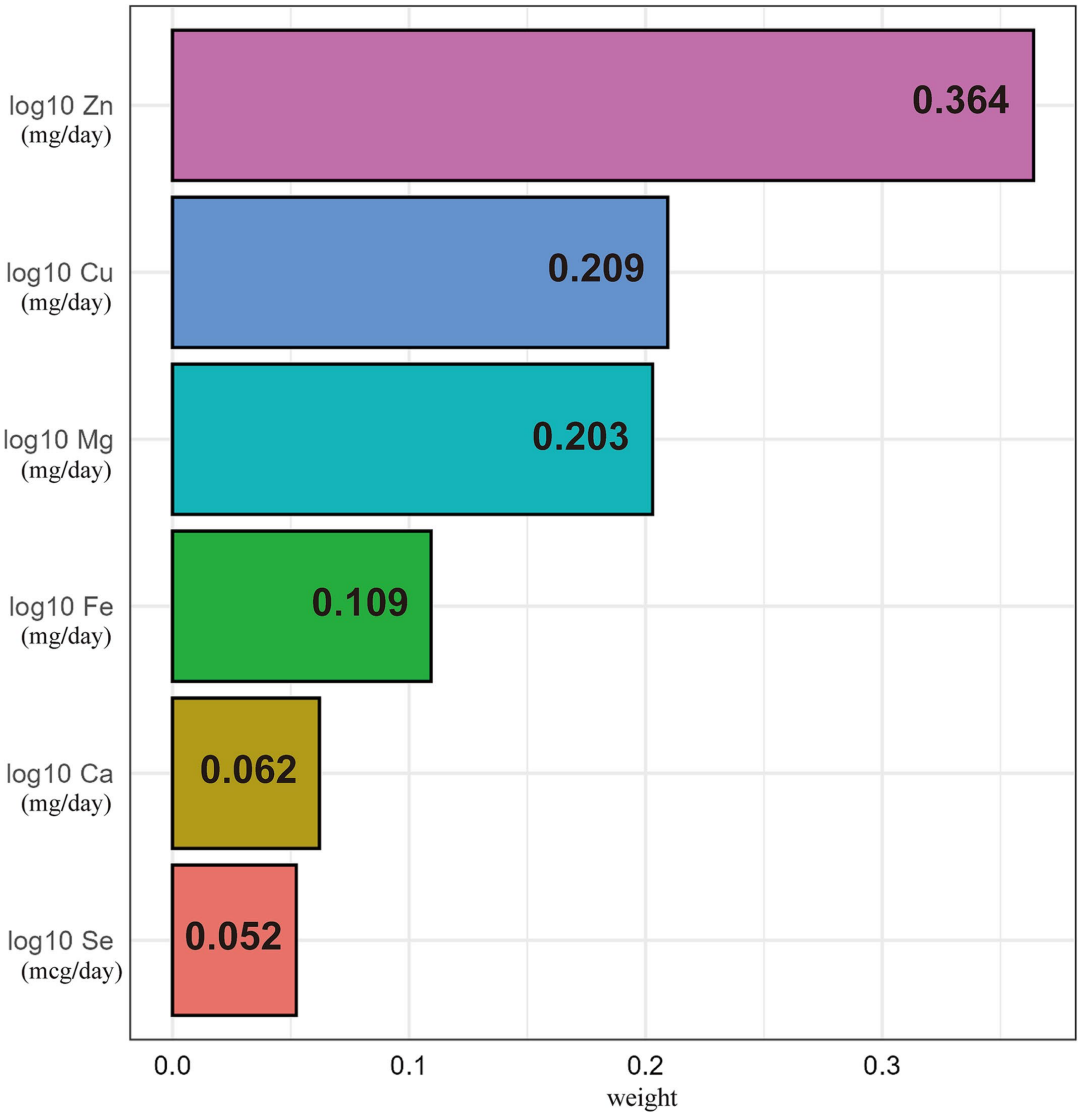
The WQS model weights of dietary metals on DR odds in negative direction with all covariates adjusted.

We applied the WQS model to examine the association between the combined effects of the six dietary metals intake and the prevalence of DR in individuals with diabetes. As shown in Table 3, the WQS index indicated that the combined effects of dietary metals intake were negatively associated with the prevalence of DR (Crude model: OR = 0.83, 95% CI 0.70–0.95, *p* = 0.005; Model 1: OR = 0.83, 95% CI 0.70–0.96, *p* = 0.008; Model 2: OR = 0.82, 95% CI 0.69–0.95, *p* = 0.008; Model 3: OR = 0.79, 95% CI 0.61–0.97, *p* = 0.024). In the fully adjusted covariates WQS regression (Figure 2), dietary Zn intake received the highest weight of 0.364 for DR risk in the negative direction, compared to weights of 0.209, 0.203, 0.109, 0.062, and 0.052 for Cu, Mg, Fe, Ca, and Se, respectively. In the positive direction, the combined effects of dietary metals intake showed no significant association with the odds of DR after fully adjusting for covariates (Crude model: OR = 0.95, 95% CI 0.83–1.06, *p* = 0.379; Model 1: OR = 0.93, 95% CI 0.79–1.06, *p* = 0.263; Model 2: OR = 0.92, 95% CI 0.78–1.05, *p* = 0.224; Model 3: OR = 0.93, 95% CI 0.75–1.12, *p* = 0.499), with dietary Zn intake receiving the lowest weight of 0.009 for DR risk, as shown in Table 3 and Supplementary Figure 1.

**Table 3:** Comprehensive impact of multi-metal intake on DR risk in diabetic population by WQS model.

WQS index	Crude model		Model 1		Model 2		Model 3	
	OR (95% CI)	*p*-value	OR (95% CI)	*p*-value	OR (95% CI)	*p*-value	OR (95% CI)	*p*-value
Positive direction	0.95 (0.83, 1.06)	0.379	0.93 (0.79, 1.06)	0.263	0.92 (0.78, 1.05)	0.224	0.93 (0.75, 1.12)	0.499
negative direction	0.83 (0.70, 0.95)	0.005	0.83 (0.70, 0.96)	0.008	0.82 (0.69, 0.95)	0.008	0.79 (0.61, 0.97)	0.024

### 3.4 Dietary metals intake and DR risk in BKMR model

**Figure 3:**
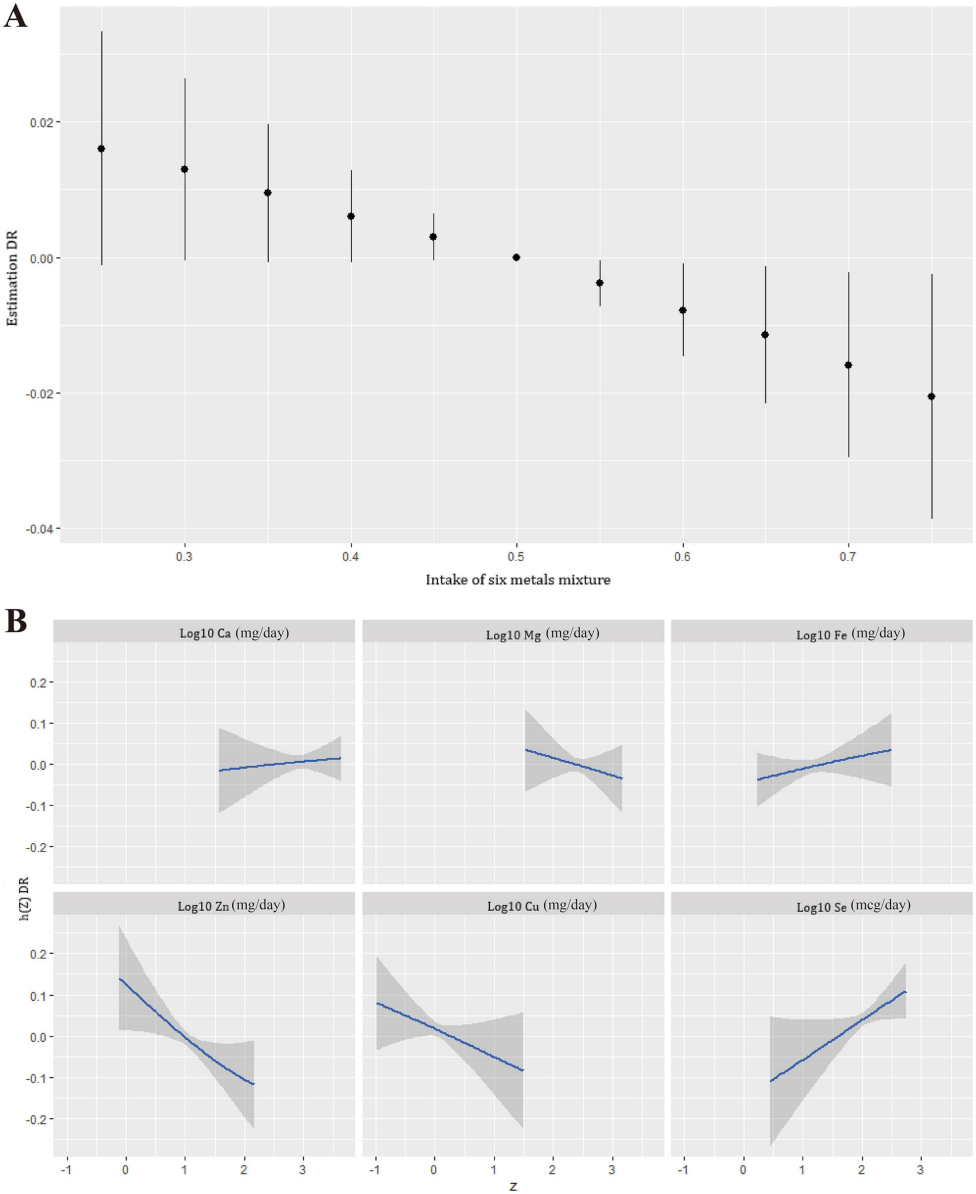
The comprehensive analysis of BKMR model on dietary metals and DR risk. **(A)** Overall effect of the six dietary metals mixture on DR risk. **(B)** Univariate exposure-response function between each dietary metal intake and DR risk when the other metal intake was simultaneously fixed at 50th percentile.

In the BKMR model, the risk of DR among individuals with diabetes was decreased for the combined intake of six dietary metals mixtures above the 50th percentile compared to the medians (Figure 3A). Figure 3B illustrated an inverse association between dietary Zn intake and the risk of DR, with the intake levels of all other dietary metals held constant at the median. Supplementary Table 1 presented a summary of the PIPs from the BKMR model. Among the dietary metals, Zn intake displayed the highest PIP (0.8574) in relation to the prevalence of DR in individuals with diabetes. Additionally, Supplementary Figure 2 possibly indicated a negative association between dietary Zn intake and DR prevalence compared to other dietary metals, when controlling for the 25th, 50th, and 75th percentiles of other dietary metals, although this association did not reach statistical significance. Furthermore, Supplementary Figure 3 indicated that there were no potential interactions among dietary metals intake.

### 3.5 Further analysis of the relationship between dietary Zn intake and DR

**Figure 4:**
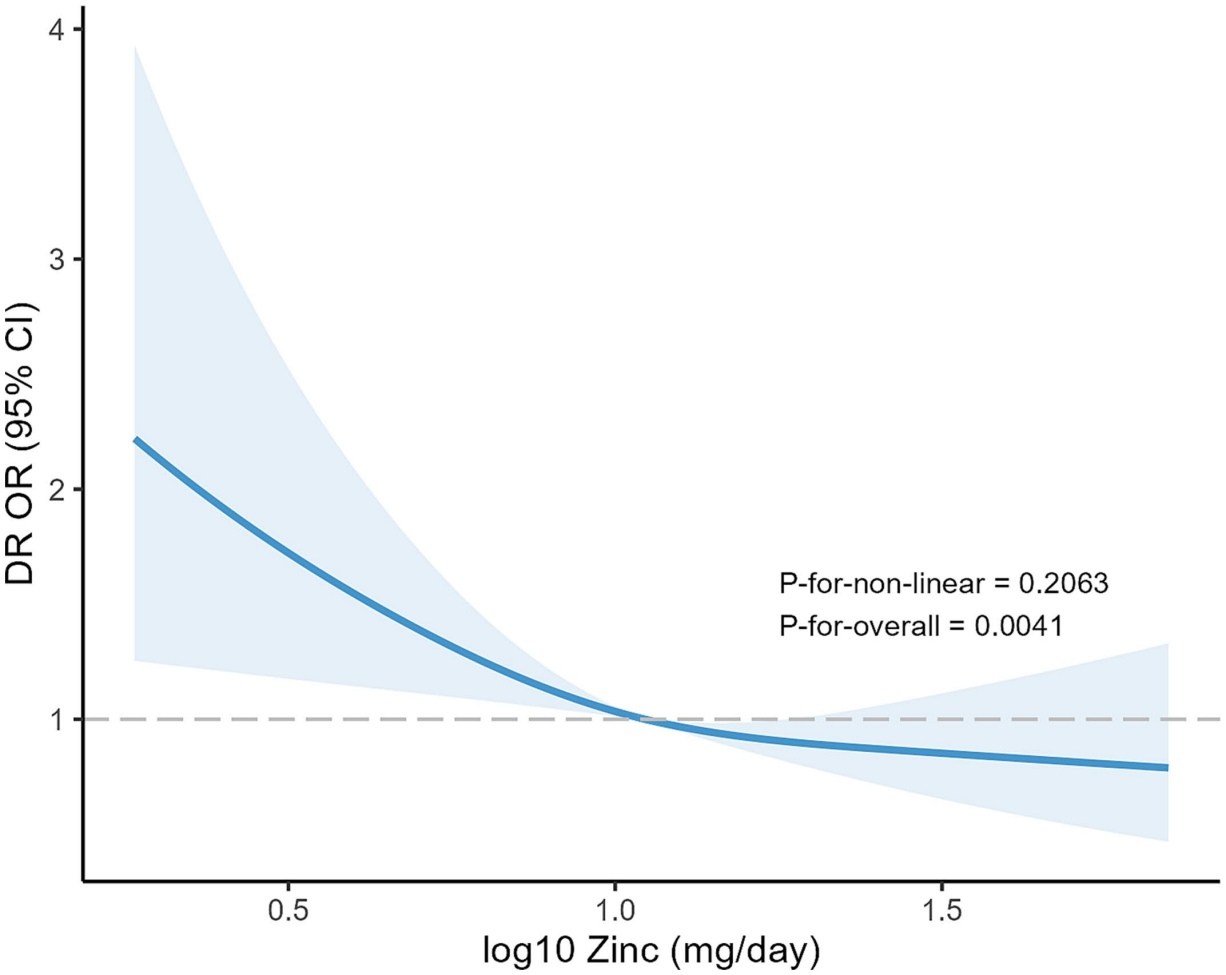
Thorough description of DR risk across the log10-transformed dietary Zn intake by RCS model.

**Figure 5:**
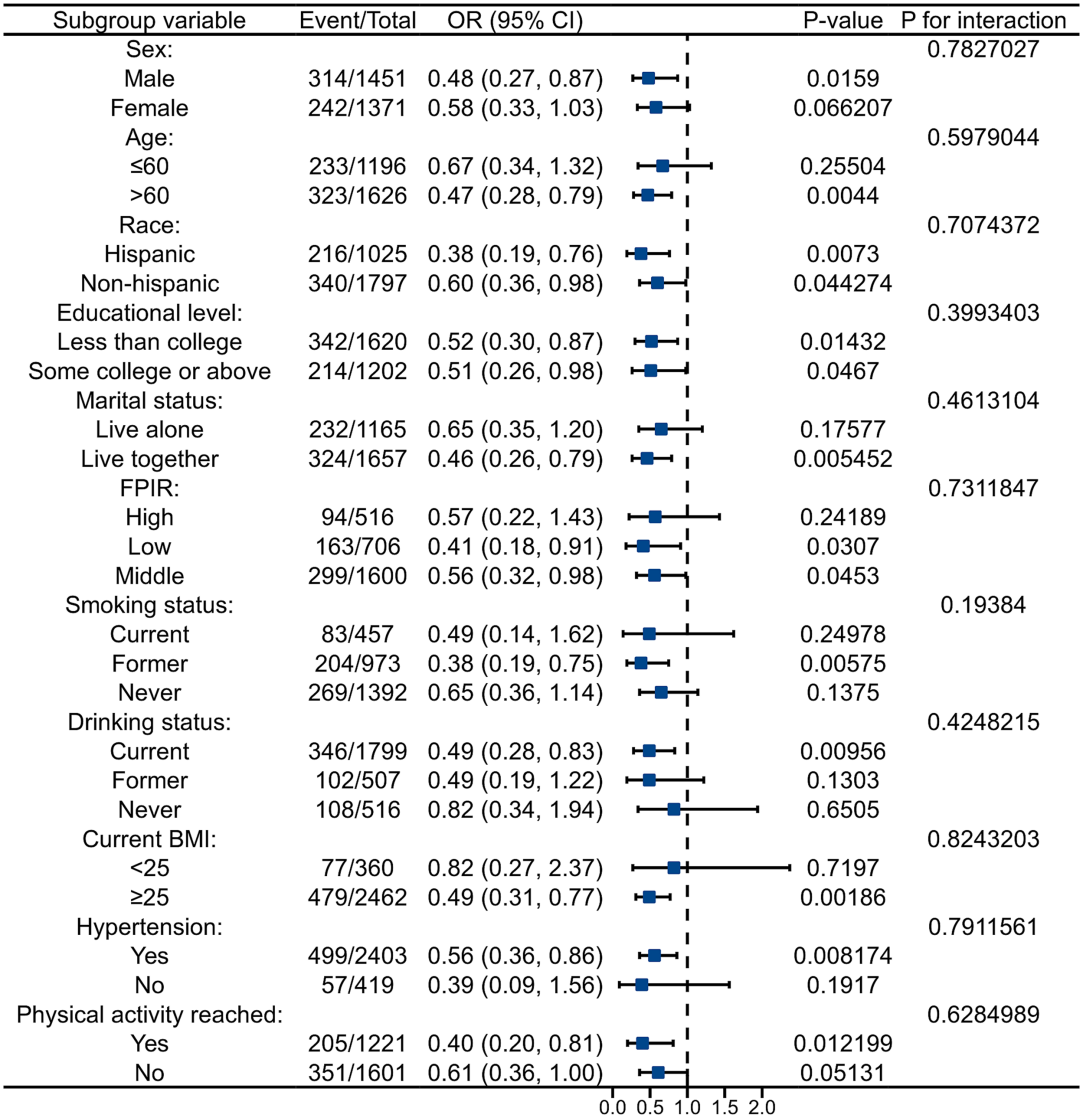
Subgroup analyses on the association of the log10-transformed dietary Zn intake with the risk of DR.

In the RCS regression model, the log10-transformed dietary Zn intake was found to have an inverse association with the risk of DR in a linear dose–response manner (*p* for non-linear = 0.2063, *p* for overall = 0.0041), as shown in Figure 4. The subgroup analysis results are presented in Figure 5, indicating that the inverse correlation between dietary Zn intake and DR was not modified by various factors such as age, sex, race, educational level, marital status, FPIR, smoking status, drinking status, BMI, hypertension and physical activity (all *p* for interaction > 0.05). As shown in Supplementary Table 2, the results of the linear analysis suggested that dietary Zn intake was significantly correlated with the concentration of serum ALB with all covariates controlled (Model 3: *β* = 2.25, 95% CI 1.33–3.82, *p* = 0.003). Additionally, the increase of serum ALB could reduce the incidence of DR in individuals with diabetes (Model 3: OR = 0.95, 95% CI 0.92–0.97, *p* < 0.001) (Supplementary Table 3). According to Table 4, the results of the mediation analysis showed that serum ALB level mediated the negative association between dietary Zn intake and DR prevalence after fully adjusting for covariates (Total effect = −0.128321, 95% CI -0.232569--0.047959, *p* = 0.002; Indirect effect = −0.008067, 95% CI -0.015724--0.002561, *p* = 0.002; Mediation proportions = 0.062866, 95% CI 0.018701–0.163004, *p* = 0.004).

**Table 4:** Mediating effect and proportions of inflammation and oxidative stress biomarkers between Zn intake and DR odds in diabetic population.

Mediator	Total effect (95% CI)	*p*-value	Indirect effect (95% CI)	*p*-value	Mediation proportions (95% CI)	*p*-value
CRP	−0.059317 (−0.169974, 0.029176)	0.218	0.000384 (−0.002569, 0.003119)	0.818	−0.006466 (−0.143667, 0.139308)	0.864
WBC	−0.123184 (−0.219131, −0.041272)	<0.001	−0.000405 (−0.002926, 0.001603)	0.678	0.003284 (−0.015910, 0.024644)	0.678
Lymphocyte	−0.124354 (−0.219582, −0.036773)	<0.001	0.000982 (−0.001114, 0.004590)	0.378	−0.007896 (−0.049456, 0.009047)	0.378
Neutrophil	−0.125031 (−0.208450, −0.041851)	<0.001	−0.000646 (−0.003198, 0.001418)	0.640	0.005169 (−0.013519, 0.028425)	0.640
ALP	−0.128417 (−0.232059, −0.042327)	0.002	−0.001847 (−0.007020, 0.000723)	0.184	0.014381 (−0.007775, 0.069986)	0.186
ALB	−0.128321 (−0.232569, −0.047959)	0.002	−0.008067 (−0.015724, −0.002561)	0.002	0.062866 (0.018701, 0.163004)	0.004
BIL	−0.128277 (−0.232943, −0.044468)	0.004	0.000741 (−0.003477, 0.005099)	0.752	−0.005775 (−0.056719, 0.031987)	0.756
GGT	−0.128013 (−0.225095, −0.047013)	<0.001	0.000027 (−0.002264, 0.001908)	1.000	−0.000212 (−0.018289, 0.023465)	1.000

## 4 Discussion

This study examined the association between dietary metals intake and the risk of diabetic retinopathy (DR) in the diabetic population aged 20 from NHANES 2007–2016. We found that higher intake of multiple dietary metals, especially zinc (Zn), was associated with lower DR risk across multiple statistical models including logistic regression, WQS regression, and BKMR. Notably, Zn was identified as the most protective metal, with a dose-dependent inverse association observed in RCS regression. Mediation analysis further revealed that serum albumin (ALB) partially mediates the negative association, suggesting a potential biological pathway.

Our findings align with prior studies reporting lower serum Zn levels in individuals with diabetes (25–28). A randomized controlled trial has confirmed that Zn supplementation can improve glycemic control in individuals with diabetes (26). Meanwhile lower serum zinc levels are associated with an increased risk of diabetic complications, suggesting a potential link between zinc status and retinal health (25, 29, 30). Additionally, a randomized controlled trial demonstrated that Zn supplementation can decelerate the progression of vision loss in individuals with macular degeneration (31). Our study further demonstrated that Zn intake is negatively associated with the risk of DR.

Zn deficiency may contribute to DR via several mechanisms, including impaired antioxidant defenses, enhanced inflammation, endothelial dysfunction, and dysregulated apoptosis—all of which are critical in DR pathogenesis (32–39). Serum ALB, the main plasma transporter of Zn (40, 41), also plays a protective role through its antioxidant and anti-inflammatory properties (42). Previous study has suggested that low serum albumin levels may increase the permeability of the optic disc and surrounding blood vessels, leading to subclinical disc edema (43). Our findings highlight the significant mediating role of albumin in the association between Zn intake and DR. The mediation effect accounted for a considerable proportion of the total effect (6.29, 95% CI 1.87–16.30%). These results suggest that albumin may be a key mechanism through which Zn deficiency contributes to the development of DR.

To the best of our knowledge, this is the first population-based study to assess the joint effects of multiple dietary metals on DR risk. By integrating multiple analytical approaches, we validated the robustness of our findings and identified Zn as a key protective component. These results may provide population-level evidence for considering Zn intake in DR prevention strategies.

However, several limitations should be acknowledged. The retrospective design precludes causal inference, and the predominantly White American sample limits generalizability. Future prospective studies in more diverse populations are needed to confirm our findings. In clinical practice, Zn supplementation could be explored as a potential preventive strategy against DR, particularly in individuals with low Zn or ALB levels.

## 5 Conclusion

Among American adults with diabetes from NHANES 2007–2016, higher dietary intake of certain metals, particularly zinc, was associated with a lower risk of diabetic retinopathy. Serum albumin may serve as a potential mediator of this protective effect.

## Data Availability

The datasets presented in this article are not readily available because the dataset employed in this study was accessed from the NCHS, accessible via the following web link: https://wwwn.cdc.gov/nchs/nhanes. Requests to access the datasets should be directed to https://wwwn.cdc.gov/nchs/nhanes.
