# Supplementary material for: Association between dietary multi-metal intake and the risk of diabetic retinopathy: a population-based study
Source: Front Nutr. 2025 Jun 19;12:1595788. doi: 10.3389/fnut.2025.1595788 (PMC12222288; doi:10.3389/fnut.2025.1595788)
Supplement: Supplementary file 1 [file Table_1.docx]

Supplementary Material

# Supplementary Figures & Tables

## Supplementary Figures

**
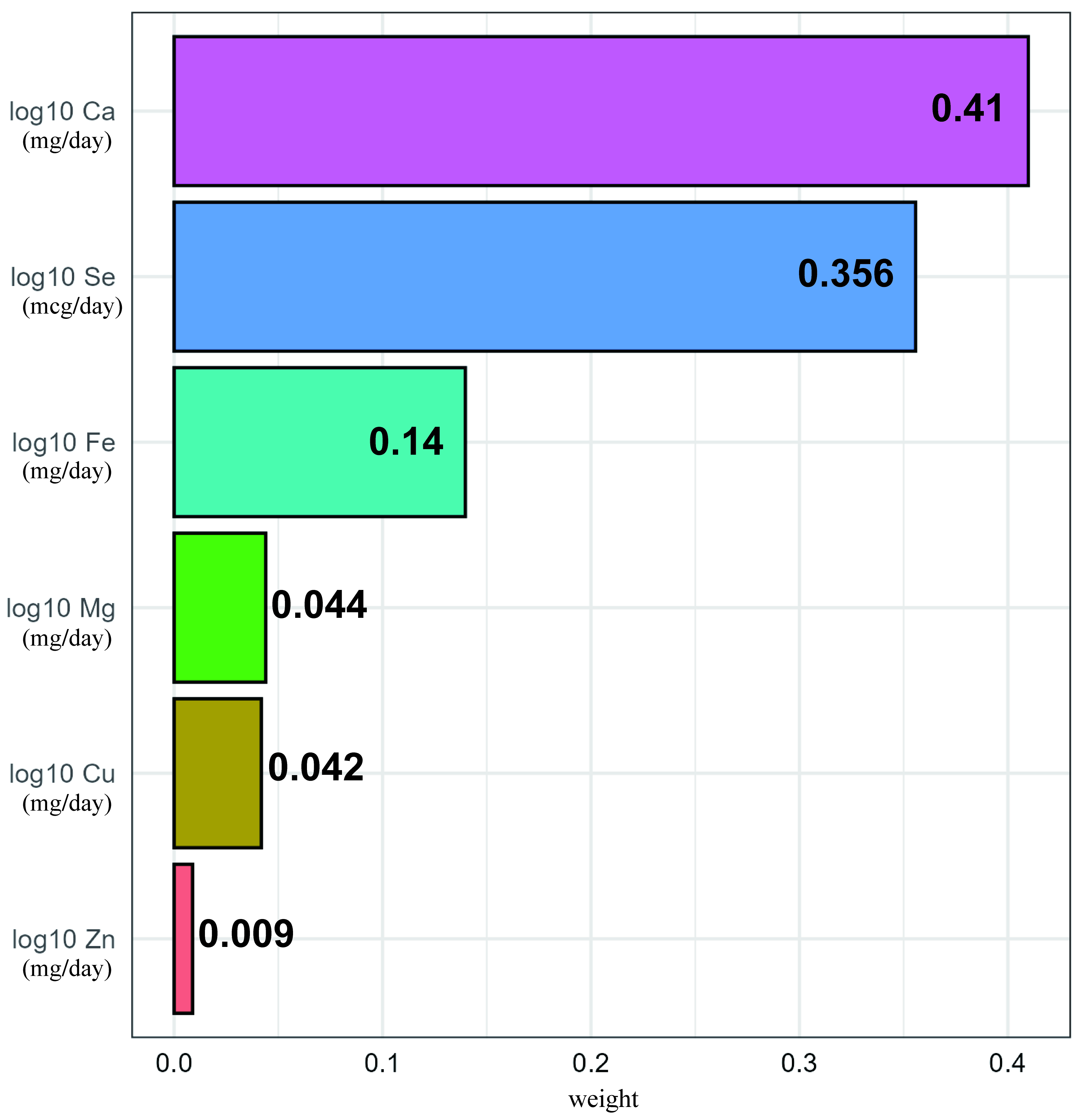
**

**Supplementary Figure 1.** The WQS model weights of dietary metals on DR odds in positive direction with all covariates adjusted.

**
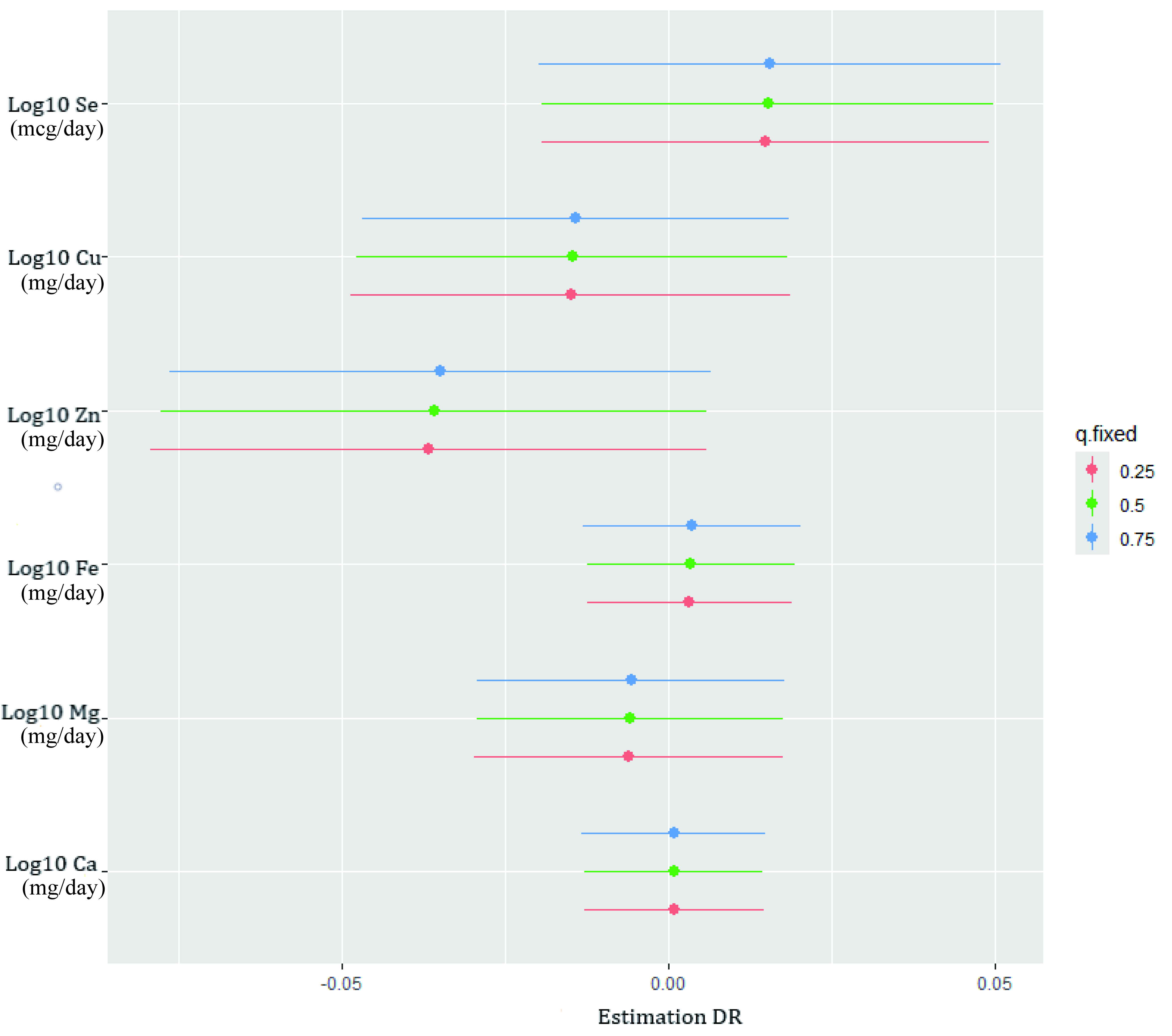
**

**Supplementary Figure 2.** The relationship between dietary metal intake and DR risk in BKMR model when other metals were simultaneously fixed at the 25th, 50th, and 75th percentiles.

**

**

**Supplementary Figure 3.** The relationship between dietary metal intake and DR risk in BKMR model when one of the other metals was fixed at the 10th, 50th, and 90th percentiles.

## Supplementary Tables

**Supplementary Table 1.** The PIPs of six metals intake in BMKR model.

| **Metal intake** | **PIPs** |
| --- | --- |
| **Zn** | 0.8574 |
| **Cu** | 0.6102 |
| **Se** | 0.6006 |
| **Mg** | 0.4788 |
| **Fe** | 0.3860 |
| **Ca** | 0.3236 |

**Supplementary Table 2.** Multivariate linear regression analysis for the relationship between Log10-transformed dietary Zn intake and mediator in diabetic population.

| **Mediator** | **Crude model** | | **Model 1** | | **Model 2** | | **Model 3** | |
| --- | --- | --- | --- | --- | --- | --- | --- | --- |
|  | **β (95% CI)** | **p-value** | **β (95% CI)** | **p-value** | **β (95% CI)** | **p-value** | **β (95% CI)** | **p-value** |
| **CRP** | 0.39 (0.10, 1.49) | 0.168 | 0.62 (0.15, 2.48) | 0.499 | 0.92 (0.24, 3.58) | 0.906 | 1.46 (0.31, 6.91) | 0.636 |
| **WBC** | 0.81 (0.60, 1.09) | 0.168 | 0.81 (0.60, 1.09) | 0.022 | 0.78 (0.58, 1.05) | 0.102 | 0.83 (0.59, 1.16) | 0.269 |
| **Lymphocyte** | 0.79 (0.69, 0.90) | <0.001 | 0.86 (0.75, 0.98) | 0.025 | 0.89 (0.78, 1.02) | 0.082 | 0.92 (0.79, 1.07) | 0.2663 |
| **Neutrophil** | 0.98 (0.78, 1.23) | 0.867 | 0.83 (0.66, 1.05) | 0.125 | 0.89 (0.71, 1.12) | 0.31246 | 0.90 (0.70, 1.16) | 0.426 |
| **ALP** | 1.27e-4 (2.01e-6, 8.08e-3) | <0.001 | 1.49e-3 (1.98e-5, 0.11) | 0.003 | 3.72e-3 (4.87e-5, 0.28) | 0.011 | 0.05 (3.97e-4, 7.27) | 0.243 |
| **ALB** | 3.65 (2.29, 5.83) | <0.001 | 2.08 (1.29, 3.35) | 0.003 | 1.95 (1.22, 3.11) | 0.005 | 2.25 (1.33, 3.82) | 0.003 |
| **BIL** | 1.95 (1.02, 3.76) | 0.045 | 0.79 (0.41, 1.54) | 0.486 | 0.63 (0.32, 1.22) | 0.171 | 0.87 (0.41, 1.86) | 0.728 |
| **GGT** | 16 (0.05, 5691) | 0.355 | 6.14 (0.01, 2785) | 0.561 | 11.8 (0.03, 5482) | 0.432 | 89.4 (0.08, 95745) | 0.207 |

**Supplementary Table 3.** Multivariate logistic regression analysis of mediator for the prevalence of DR in diabetic population.

| **Mediator** | **Crude model** | | **Model 1** | | **Model 2** | | **Model 3** | |
| --- | --- | --- | --- | --- | --- | --- | --- | --- |
|  | **OR (95% CI)** | **p-value** | **OR (95% CI)** | **p-value** | **OR (95% CI)** | **p-value** | **OR (95% CI)** | **p-value** |
| **CRP** | 1.01 (0.99, 1.02) | 0.421 | 1.01 (0.99, 1.02) | 0.375 | 1.01 (0.99, 1.02) | 0.414 | 1.01 (0.99, 1.02) | 0.454 |
| **WBC** | 1.00 (0.96, 1.05) | 0.837 | 1.02 (0.98, 1.06) | 0.389 | 1.02 (0.97, 1.06) | 0.431 | 1.01 (0.97, 1.06) | 0.582 |
| **Lymphocyte** | 0.93 (0.83, 1.03) | 0.219 | 0.95 (0.84, 1.05) | 0.364 | 0.94 (0.84, 1.05) | 0.329 | 0.94 (0.83, 1.05) | 0.323 |
| **Neutrophil** | 1.02 (0.97, 1.08) | 0.429 | 1.04 (0.99, 1.10) | 0.142 | 1.04 (0.98, 1.10) | 0.148 | 1.03 (0.98, 1.10) | 0.249 |
| **ALP** | 1.00 (1.00, 1.01) | 0.008 | 1.00 (1.00, 1.01) | 0.007 | 1.00 (1.00, 1.01) | 0.009 | 1.00 (1.00, 1.01) | 0.019 |
| **ALB** | 0.95 (0.92, 0.97) | <0.001 | 0.94 (0.92, 0.97) | <0.001 | 0.94 (0.92, 0.97) | <0.001 | 0.95 (0.92, 0.97) | <0.001 |
| **BIL** | 0.97 (0.95, 0.99) | 0.015 | 0.97 (0.95, 0.99) | 0.004 | 0.97 (0.95, 0.99) | 0.005 | 0.97 (0.95, 0.99) | 0.007 |
| **GGT** | 1.00 (1.00, 1.00) | 0.704 | 1.00 (1.00, 1.00) | 0.829 | 1.00 (1.00, 1.00) | 0.954 | 1.00 (1.00, 1.00) | 0.968 |
